# Functional characterization of the MED12 p.Arg1138Trp variant in females: implications for neural development and disease mechanism

**DOI:** 10.1186/s10020-025-01365-5

**Published:** 2025-09-29

**Authors:** Nicole C. Shaw, Saraya Harrison, Kevin Chen, Catherine A. Forbes, Emma Kuzminski, Mitchell Hedges, Kathryn O. Farley, Michelle Ward, Lily Loughman, Cathryn Poulton, Gareth Baynam, Timo Lassmann, Vanessa S. Fear

**Affiliations:** 1 The Kids Research Institute Australia, The University of Western Australia, Nedlands, WA Australia; 2 School of Medical, Molecular and Forensic Sciences, Murdoch University, Murdoch, WA Australia; 3 Western Australian Register of Developmental Anomalies, King Edward Memorial Hospital, Undiagnosed Diseases Program, Genetic Health of WA, Subiaco, WA 6008 Australia; 4 Rare Care Centre, Perth Children’s Hospital, Nedlands, WA 6009 Australia

**Keywords:** MED12, Rare disease, Patient-derived iPSCs, Human neural disease model, Functional genomics, Mediator complex, Ribogenesis

## Abstract

**Background:**

Seven female individuals with multiple congenital anomalies, developmental delay and/or intellectual disability have been found to have a genetic variant of uncertain significance in the mediator complex subunit 12 gene (*MED12* c.3412C>T, p.Arg1138Trp). The functional consequence of this genetic variant in disease is undetermined, and insight into disease mechanism is required.

**Methods:**

We identified a *de novo* MED12 p.Arg1138Trp variant in a female patient and compared disease phenotypes with six female individuals identified in the literature. To investigate affected biological pathways, we derived two induced pluripotent stem cell (iPSC) lines from the patient: one expressing wildtype MED12 and the other expressing the MED12 p.Arg1138Trp variant. We performed neural disease modelling, transcriptomics and protein analysis, comparing healthy and variant cells.

**Results:**

When comparing the two cell lines, we identified altered gene expression in neural cells expressing the variant, including genes regulating RNA polymerase II activity, transcription, pre-mRNA processing, and neural development. We also noted a decrease in *MED12L* expression. Pathway analysis indicated temporal delays in axon development, forebrain differentiation, and neural cell specification with significant upregulation of pre-ribosome complex gene pathways.

**Conclusion:**

In a human neural model, expression of MED12 p.Arg1138Trp altered neural cell development and dysregulated the pre-ribosome complex providing functional evidence of disease aetiology and mechanism in MED12-related disorders.

**Supplementary Information:**

The online version contains supplementary material available at 10.1186/s10020-025-01365-5.

## Introduction

Genetic variants in *MED12* are linked to various rare syndromic disorders. However, there is limited understanding of the specific pathways through which *MED12* variants contribute to disease pathology. We investigate a *MED12* c.3412 C > T, p.Arg1138Trp variant located on the X chromosome, identified in seven female individuals with severe congenital abnormalities and developmental delays. MED12 is part of the Mediator complex and links transcription factors with RNA polymerase II, modulating their function and activity, respectively [1].

The Mediator complex in humans is 1.4 MDa, comprising 26 subunits, and is responsible for the association of RNA Polymerase II with DNA-binding transcription factors and formation of the pre-initiation complex [2]. *MED12* encodes the Mediator subunit 12, and in complex with MED13, Cyclin C, and CDK8, forms the Mediator Kinase Module (MKM). The MKM is involved in phosphorylation of transcription factors, which prevents association of the Mediator complex with POLR2A [1]. MED12L and MED13L are mutually exclusive with MED12 and MED13, respectively, in MKM formation. In addition, CDK19 acts as a paralog of CDK8, which adds complexity to the understanding of the Mediator complex. During development, the Mediator complex is involved in epigenetic modification, formation of super-enhancers [1, 3], and epigenetic silencing of neuronal gene expression [4].

A number of syndromes are linked to mutations in *MED12* [5–7] including FG syndrome, type 1 (FGS1, or Opitz-Kaveggia syndrome); Lujan syndrome (LS, Lujan-Fryns syndrome, or intellectual disability, X-linked, with marfanoid habitus); X-linked Ohdo syndrome (XLOS; also called blepharophimoisis – intellectual disability syndrome, Maat-Kievit-Brunner (MKB) type) [8]; and Hardikar Syndrome (also known as cholestasis-pigmentary retinopathy-cleft palate syndrome). Additionally, particular *MED12* variants do not always correspond to specific syndromes and are instead classified as MED12 non-specific intellectual disability [9]. The majority of the MED12-related syndromes were initially identified as hemizygous variants in males. However, diagnosis in females presenting with clinical features of disease may be dependent on X-chromosome inactivation skewing [9].

In this study, we perform the first functional analysis of the *MED12* c.3412 C > T, p.Arg1138Trp variant in patient-derived iPSCs (healthy and variant-expressing iPSCs), using neural disease modelling, transcriptomics, and protein analysis. The study identifies key changes in the transcription machinery following *MED12* variant expression in neural cells. The changes include both temporal alterations in neural development and dysfunction in ribosome biogenesis. Additionally, we provide an overview of MED12 syndrome-related phenotypes, specifically comparing the clinical phenotype across seven female individuals with the *MED12* c.3412 C > T, p.Arg1138Trp variant.

## Materials and Methods

### Patient genome sequencing

The Department of Diagnostic Genomics (Pathwest, Western Australia) confirmed this patient to be heterozygous for the *MED12* missense variant of uncertain significance NM_005120.2(MED12) c.[3412 C > T]; [3412=] p.[(Arg1138Trp)]; [(Arg1138=)] by Sanger sequencing of gDNA, which confirmed previous Whole Genome Sequencing by Genome One (ref. SYD-40669926). The variant was classified as a variant of uncertain significance (VUS) according to ACMG guidelines [10], and the *MED12* variant was confirmed as *de novo*. The Normalizer description for the *MED12* variant is GRCH38 NC_000023.11(NM_005120.2): c.3412 C > T; NC_000023.1: g.71,128,655 C > T; NM_005120.2(NP_005111.2) p.(Arg1138Trp) and is herein referred to as MED12_VUS.

### Patient-derived iPSCs

A 5 mL blood sample was collected from the patient into EDTA Vacutainer tubes, with PBMCs prepared using Lymphoprep (Stem Cell Technologies) and cryopreserved in Freezing Media (Stem Cell Technologies). PBMCs were thawed in RPMI/10% FCS and expanded in PBMC medium (CTS StemPro HSC Basal Medium with CTS StemPro HSC Supplement, 100ng/mL SCF, 100ng/mL FLT-3, 20ng/mL IL-3 and 20ng/mL IL-6). PBMCs were reprogrammed using the CytoTune-iPS Sendai 2.1 Reprogramming Kit with KOS (MOI = 5), hc-Myc (MOI = 5), and hKlf4 (MOI = 3) reprogramming vectors (Invitrogen). Emerging iPSC colonies were manually picked under sterile conditions and serially passaged. Next, RNA was extracted (RNeasy Minikit, Qiagen) from each iPSC clone and assessed for the presence of transgenes by RT-qPCR to confirm viral clearance. Briefly, cDNA was generated using Superscript IV Reverse Transcriptase according to the manufacturer’s instructions. The expression of viral genes was then assessed (Suppl. Figure 1 A) using commercially available Taqman assays for SeV (Mr04269880_mr), KOS (Mr04421257_mr), Klf4 (Mr04421256_mr) and cMyc (Mr04269876_mr), with GAPDH (Hs99999905_m1) as the endogenous control, and the Taqman Fast Advanced Master Mix (Applied Biosystems) using the QuantStudio 7 Flex system (Applied Biosystems).

### Karyotyping

Genomic DNA samples from all iPSC Lines were screened for 8 common karyotypic abnormalities (Suppl. Figure 1 C) reported in human embryonic stem cells and iPSCs using the hPSC Genetic Analysis Kit (STEMCELL Technologies) according to manufacturer’s instructions, using the QuantStudio Flex system (Applied Biosystems) at cycling conditions specified by the hPSC Genetic Analysis kit [11].

### Trilineage differentiation

iPSC clones were stimulated for differentiation to all three germ layers using the STEMdiff Trilineage Differentiation Kit according to the manufacturer’s instructions. At day 0, day 5 (endoderm and mesoderm) and day 7 (ectoderm) of differentiation, cells were harvested and assessed for tissue markers by flow cytometry (Suppl. Figure 1D). Cells were stained with live-dead dye FVS780 and primary antibody CXCR4-PECy7 (12G5; eBioscience) prior to fixation and permeabilization using the eBioscience FOXP3/Transcription Factor Staining Buffer Set. Cells were subsequently stained with primary antibodies OCT-3/4-AF488 (40/Oct-3; BD), SOX17-PerCP-Cy5.5 (P7-969; BD), Brachyury-APC (R&D Systems), PAX6-PE (O18-1330; BD), and Nestin-AF594 (10C2; BioLegend), and assessed on an LSRII Fortessa (Becton Dickinson). Changes in fluorescence intensity of lineage markers between iPSCs and germ layers with respect to isotype controls were analysed in FlowJo (v10.7.2).

### Cell culture

iPSCs were grown in 6-well plates coated with Matrigel, hESC-qualified matrix, LDEV-free (Corning) in TeSR-E8 (STEMCELL Technologies). Media were changed daily and cultures monitored for stem cell morphology by Light microscopy. Cells were dissociated with Gentle Cell Dissociation Reagent and cultured with 10 µM ROCK Inhibitor (Y-27632, STEMCELL Technologies) for 24 h after re-plating. All cultures were grown in humidified conditions at 37 °C in a 5% CO_2_ incubator. Cell cryopreservation was in Knock-Out Serum Replacement with 10% DMSO (Gibco). Derived cell lines were of normal stem cell morphology (Suppl. Figure 1B).

### Confirmation of the MED12 genetic variant in patient-derived iPSC lines

To confirm the presence of the MED12_VUS in the patient-derived clonal iPSC Lines, 252 bp PCR amplicon products across the MED12_VUS site were prepared from gDNA with primers MED12-pTSF1 (5’ACACTCTTTCCCTACACGACGCTCTTCCGATCTACACTGAGTCATGGTGTCTGTC3’) and MED12-pTSR1 (5’GTGACTGGAGTTCAGACGTGTGCTCTTCCGATCTAGGTCTCCCACAGTGAACAA3’) and Phusion High-Fidelity PCR Master Mix (Thermo Scientific) [12–14]. Samples were AMPure XP Bead purified (Agencourt) and PCR2 barcoded with TruSeq primers (IDT) for paired-end, 250 bp sequencing with a MiniSeq Mid-Output Kit, 300 cycles. Reads were analysed with CRISPResso2 software [15]. Additionally, 247 bp amplicon products across the MED12_VUS site were prepared from cDNA with primers MED12_cDNA pTSF1 (5’ACACTCTTTCCCTACACGACGCTCTTCCGATCTGCCTTGTGCTGCTCCTCTAA3’) and MED12_cDNA pTSR1 (5’GTGACTGGAGTTCAGACGTGTGCTCTTCCGATCTAAAGGTGAAGGAGGATGCGG3’) to detect expression of the MED12_WT or MED12_VUS using amplicon sequencing and CRISPResso2 analysis.

### Neural disease modelling

iPSCs were stimulated to differentiate into neural progenitor cells (NPCs) with STEMdiff SMADi Neural Induction Kit (STEMCELL, Vic, Australia) [11, 13, 14]. At the indicated time points, 5 × 10^5^ cells were collected, fixed/permeabilized with transcription factor staining buffer set (eBioscience, US), and live/dead stained with FVS780 (Becton Dickinson). Cells were then stained with primary antibodies for stem cell expression markers with OCT3-AF488 (40/Oct-3; BD), and NANOG-BV421 (16H3A48; BioLegend); and neural markers with PAX6-PE (O18-1330; BD) and Nestin-AF647 (10C2; BioLegend) and then analysed on an LSRII Fortessa (Becton Dickinson). Cell marker expression was reported as a percentage of live cells. Three independent neural differentiations of MED12_WT and MED12_VUS iPSC lines were performed at different times and separate passages to derive the biological replicates (*n* = 3) required for statistical analysis. Statistical analysis of cell marker expression was performed with two-way ANOVA and Bonferroni’s correction for multiple comparisons using GraphPad Prism. In addition, cells were collected for RNA extraction (RNeasy Minikit, with DNase on column treatment; Qiagen) from each independent neural differentiation.

### MED12 western blot and immunohistochemistry

Proteins were extracted from cells lysed with Pierce’s co-IP protein lysis buffer (ThermoFisher Scientific), quantified (Direct-Detect ^®^ Infrared Spectrometer, Merck Millipore), electrophoresed on NuPAGE Tris-Acetate 3–8% protein gels (Life Technologies), and transferred to Polyvinylidene fluoride (PVDF) membranes (Life Technologies). The HiMark protein standard (LC568, Invitrogen) was used. Membranes were blocked overnight at 4 °C with Intercept^®^ (TBS) Blocking Buffer (LI-COR Biosciences), then incubated with rabbit anti-human MED12 monoclonal antibody (1:1000; clone BLR084G; Bethyl Laboratories, USA) and/or β-actin antibody (1:2000; MA5-15729; Life Technologies, Australia). After washing, membranes were probed with goat anti-rabbit IRDye^®^ 800CW or goat anti-mouse IRDye^®^ 680RD secondary antibodies (both 1:10,000; LI-COR Biosciences), and images were captured on an Odyssey Infrared Imaging System (LI-COR Biosciences). Protein expression levels were normalised to β-Actin [16].

iPSCs or NPCs were cultured on Matrigel-coated chamber slides (ibidi), fixed with 3.7% formaldehyde (Sigma-Aldrich) for 20 min, permeabilised for 15 min with 0.1% Triton-X-100 (Sigma-Aldrich), and blocked with Intercept^®^ Blocking Buffer (LI-COR). Cells were incubated overnight (4 °C) with rabbit polyclonal anti-MED12 antibody (1:250; Bethyl Laboratories, USA). Primary antibody stains were washed in 0.05% Tween-20 (Sigma-Aldrich)/PBS and incubated with either Alexa-Fluor 488-conjugated anti-rabbit antibody (1:1000; Invitrogen) or Alexa-Fluor 568-conjugated anti-mouse antibody (1:1000, Invitrogen) for 1 h at room temperature. Nuclei were stained using NucBlue stain (Invitrogen) according to the manufacturer’s instructions. Cell stains were visualised on a Nikon Eclipse TS2R inverted fluorescence microscope, and images were captured with a monochrome DS-Qi2 camera (Nikon). Immunofluorescence images were processed using NIS-Elements software (v.5.21.00) and ImageJ (v.1.54f). A negative control lacking primary antibody was used to set the background fluorescence, and all images were processed using identical LUTs.

### RNA sequencing

RNA integrity was high (RIN > 9.6), as determined with RNA ScreenTape (Eukaryotic Analysis, Genomics WA, Perth, Australia). RNA sequencing was performed using the SureSelect Strand-Specific RNA Library Preparation protocol for Illumina Multiplexed Sequencing. Libraries were sequenced on a NovaSeq 6000 platform (Illumina, USA) at Genomics WA (Perth, Australia) to a depth of approximately 30 million 100-bp paired-end reads per sample.

### Transcriptomic analysis

#### Pre-processing, exploratory data analysis and differential analysis

Processing of raw sequencing reads, differential gene expression analysis, and enrichment analyses were performed as previously described [11]. Enrichment analyses were performed using GSEA functions embedded in the clusterProfiler (v.4.8.2) [17] and DOSE (v.3.26.1) [18] packages, querying against the DisGeNET, Disease Ontology (DO) and Gene Ontology (GO) databases. The full analysis script is available in the Supplemental Experimental Procedures MED12_Analysis Script.

#### Comparison to publicly available RNAseq data

We extracted RNAseq data from wild-type NPCs in ARCHS4 (version 2.1) using h5read [19, 20], filtered genes, fitted limma-trend models, and generated PCA plots [14].

### Patient recruitment

Patient contact was initiated by a genetic counsellor at Genetic Health of Western Australia, and written informed consent was obtained by a study member. The study was in adherence with the Declaration of Helsinki and the NHMRC National Statement on Ethical Conduct in Human Research, as approved by the Child and Adolescent Health Services, Human Research Ethics Committee, RGS000000166.

## Results

### Identification of a MED12 variant of uncertain significance

**Fig. 1:**
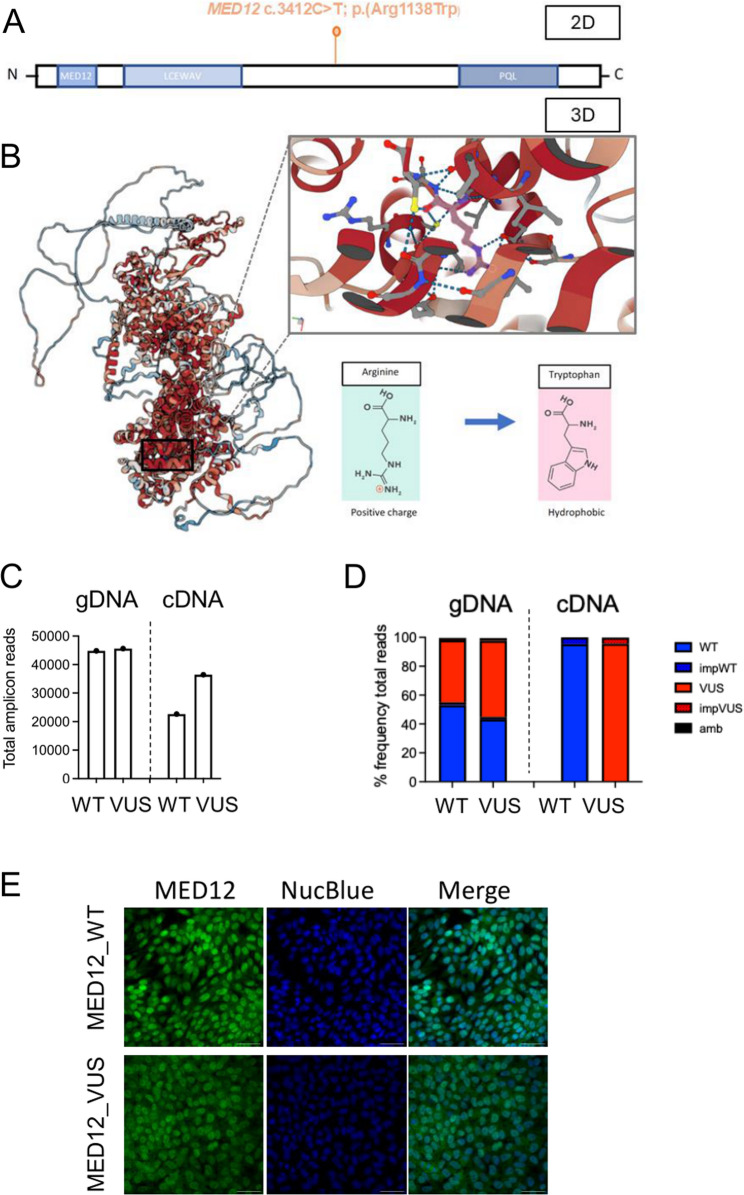
MED12 patient-derived iPSCs and protein expression.** A** Schematic indicating the *MED12* variant location and known protein domains. **B** MED12 3D modelling utilizing AlphaMissense indicating position and change of amino acid. **C and D** Targeted amplicon sequencing of gDNA and cDNA showing read counts and the percentage of reads aligning to *MED12* WT or *MED12* VUS. **E** Immunofluorescent staining of the variant and WT iPSCs indicating cellular localization of MED12 protein. White bar, 60 μm

We identified a heterozygous missense variant in *MED12* c.3412 C > T; p.Arg1138Trp on the X chromosome, as a *de novo* variant in a four-year-old female (Patient 1) with multiple congenital anomalies and developmental delay. This variant (MED12_VUS) is absent from the gnomAD control database and lies outside the three characterised domains, the MED12, LCEWAV, and the PQL domain (Fig. 1A). AlphaMissense [21] classified the variant as “Likely Pathogenic” with a score of 0.9934 (scale 0–1; higher values indicate greater pathogenicity).

The clinical geneticist reported 77 Human Phenotype Ontology (HPO) terms for the MED12 p.Arg1138Trp Patient 1 (Suppl. Table 1), which were compared to the 265 terms linked to MED12-related disorders in the HPO database (Suppl. Table 2). Thirty-one HPO terms overlapped between Patient 1 and MED12-related disorders: 16 with Ohdo X-linked (XLOS), 15 with FG syndrome (FGS), and 10 with Lujan-Fryns syndrome. Only two terms, failure to thrive and patent ductus arteriosus overlapped with Hardikar syndrome (HS).

A literature review identified six additional females (Individuals 1–6; Table 1) harbouring the same variant [22–24]. Their diagnoses varied: Blepharophimosis intellectual disability syndrome MKB type, cholestasis pigmentary retinopathy cleft palate syndrome, and other MED12-related conditions, but none were classified as FG, Lujan, or Ohdo syndrome. Across all seven individuals, common features included severe intellectual disability; behavioural abnormalities such as autism, social difficulties, poor attention span, and global developmental delay; ophthalmologic anomalies; and skull anomalies. Other frequent findings were gastrointestinal issues (constipation, dysmotility, vomiting), delayed or absent speech, and cardiac defects, with occasional skeletal (hand and foot), eyelid (ptosis), and ear anomalies.

**Table 1:** Comparison of females with the MED12 Arg1138Trp variant

Individual	Patient 1	Individual 1	Individual 2	Individual 3	Individual 4	Individual 5	Individual 6
developmental/neurological features							
walking at age	>2.5 y	NA	NA	NA	sitting at 30 months; absent ambulation at 8.5y	NA	narrow forehead; frontal bossing
speech abilities	mostly non-verbal	absent speech (5 y)	short sentences	delayed	severely delayed; no speech (8.5y)	NA	absent speech
intellectual disability (IQ)	severe	severe	severe	severe	severe	Yes	severe
behavioral abnormalities	autism; depression; obsessive compulsive behaviour; aggressive behaviour; abnormal social behaviour	autism spectrum disorder; severe sleep disorder	autistic features	Yes; developmental delay; hyperphagia; emesis	social difficulties	autistic behaviour; short attention span	Global developmental delay;
seizures	yes	no	no	NA	tonic-clonic seizures at 3y, sleep disorder	NA	NA
muscular hypotonia	yes	yes	no	Yes	NA	NA	yes,
MRI anomalies	At 5 y. Mild prominence bifrontal extra-axial SCF space; suggested cerebral volume loss; widenned foramen of Magendie; increased tegmento-vermian angle	thinning of cerebral cortex	NA	normal	delayed myelination; thick cortex; abnormal cortical gyration; ventricular dilatation	NA	NA
growth							
age of gestation	38 wks	39 wks	41 wks	40wks	39 WA + 4D	NA	Premature birth
weight at birth (SD)	−0.67 SD	2270 g (<−2.5)	3330 g (−0.58)	1800 g (z score −5.39), <10th percentile	2860 g; normal range	NA	NA
length at birth (SD)	+1.25 SD	NA	50 cm (−1)	NA	45.5 cm (<3rd percentile)	NA	NA
head circumference at birth (SD)	+1.2 SD	36.2 cm (0) (4 wks)	small for gestational age	NA	31 cm (<3rd percentile)	NA	NA
length at last investigation (SD)	−2 SD (at 2.5 years of age)	NA	154 cm (<−2.5)	NA	−3.0 SD	NA	NA
head circumference at last investigation (SD)	0 SD (at 2.5 years of age)	NA	54 cm (+1)	NA	−2.5 SD	NA	NA
obestity/BMI	no			obese, 51.1 kg/m2	−2.5 SD	NA	NA
skeletal anomalies							
syndactyly	no	fingers 3/4 subtotal	toes 2/3 (cutaneous), R>L	no	left fingers 3/4; bilateral toes 2/3	NA	toes 2/3; syndactyly finger 3/4
other finger/hand anomalies	tapered finger; clinodactyly toes 4/5	finger hyperextensability	small hands	no	bilateral camptodactyly	brachydactyly; clinodactyly	Clinocactyly of the 5th finger; postaxial hand polydactyly; Aplasia/Hypoplasia of the distal phalanges of the hand; hyperconvex nail;
scoliosis/pectus anomalies	rib fusion	NA	NA	no	NA	NA	NA
other skeletal anomalies	short foot; butterfly vertebrae; slender long bone; reduced bone mineral density	no	small feet	no	NA	NA	pes planus; sandal gap
other anomalies							
ophthalmologic/visus	strabismus;	strabismus, nystagmus	hyperopia	strabismus; estropia	NA	NA	NA
anal anomalies	no	no	no	NA	normal	NA	NA
cardiac anomalies	PDA	PDA	NA	none	Tetralogy of Fallot	Ventricular septal defect	echogenic intracardiac focus
feeding difficulties	ankyloglossia; Failure to thrive, gastric dysmotility.	no	tube feeding as neonate	no	NA	NA	ankyloglossia reported
laryngotracheomalacia or dysplasia/tracheostoma	Hip dysplasia; pulmonary hypoplasia	NA	NA	no	NA	NA	NA
recurrent infections	Yes	NA	NA	NA	NA	NA	NA
hemangioma/capillary anomalies	hemothorax; congential diaphragmatic hernia; aplasia of the left hemidiaphragm	yes (forehead)	NA	NA	NA	NA	NA
hearing loss	hearing impairment	NA	NA	NA	conductive hearling loss with abnormal temporal bone CT scan	NA	NA
skin anomalies	no; icthyosis	NA	NA	Acanthosis nigricans in facial folds	angioma; no pigmentation anomalies; left cheek tag†	NA	NA
other anomalies	gastrointestinal dysmotility; ketotic hypoglydemia; malposition of the stomach; volvulus; bilateral coxa valga; scarring; hirsutism	no	lateral neck fistula left, irritable bowel syndrome, chronic cystitix, constipation	recurrent vomiting	tendency to constipation	NA	hydronephrosis; sparse hair; chronic constipation
facial dysmorphisms							
facial shape	square face; midface retrusion	normal	normal, coarsening	course facial features	NA	NA	
skull anomalies	prominent forehead; flat forehead; microcephaly; brachycepahly, plagiocephaly; relative macrocephaly	lambdoid synostosis	large fontanel, prominent forehead, bitemporal narrowing	microcephaly	NA	NA	narrow forehead; frontal bossing
hypertelorism	yes	no	no	NA	NA	NA	yes
telecanthus	no	no	no	NA	NA	NA	yes
epicanthus	no	no	no	NA	bilateral epicanthus	NA	NA
downslanting palpebral fissures	no	no	no	NA	NA	NA	NA
upslanting parebral fissures	yes	NA	NA	Yes	NA	NA	NA
ptosis	bilateral ptosis	yes	no	no	exopthalmia with right ptosis	NA	Congenital bilateral ptosis
blepharophimosis	no; bilateral variable ptosis from birth	no	no	NA	NA	defined in condition report	NA
palatal anomalies	bifid uvula;	no	NA	NA	NA	NA	high palate
dental	no	no	no	NA	NA	NA	widely spaced teeth
mandibular	Yes, micrognathia	no	no	NA	pointed chin	NA	prognathism
ear anomalies	posteriorly rotated ears	no	small, dysplastic, low set, posteriorly rotated, auricular tag left	NA	low set posteriorly rotated ears;bilateral preauricular ear tag; right preauricular pit	NA	posteriorly rotated ears
other facial dysmorphism	microdontia; thin upper lip vermillion; narrow mouth; anteverted naresl	no	low hanging columella	NA	thin lips	NA	wide mouth; broad philtrum; bulbous nose;
Other							
placenta	Polyhydramnios	NA	NA	abnormal	single umbilical artery in 3rd trimester	NA	Meconium stained amniotic fluid
other	NA	NA	NA	Polycystic ovary syndrome (PCOS), pre-diabetes	NA	NA	Abnormal delivery, C-section, secondary caesarian section,
Described condtion	MED12-related condition	Choleostasis-pigmentary retinopathy-cleft palate syndrome	Choleostasis-pigmentary retinopathy-cleft palate syndrome	phenotype does not resemble FG, Lujan or Ohdo syndrome	MED12 related disorder	Blepharophimosis-intellectual disability syndrome, MKB type	MED12-related condition
Classification	Uncertain significance, upgraded to pathogenic (Sept, 2024)	Likely pathogenic; no functional studies	Likely pathogenic; no functional studies	Likely Pathogenic, no functional analysis	unclassified	Likely pathogenic	Uncertain significance.A15:H68
PMID	-	33244165, Polla et al.	33244165, Polla et al.	33913598, Gonzalez et al.	34079076, Riccardi et al	-	-
Clinvar accession		SCV003841805.1, Mar 18, 2023	SCV003841805.1, Mar 18, 2023	none		SCV002577676.1, Oct 08, 2022	SCV002098350.1, Feb 26, 2022
Variant/condition record	Precision Medicine study	href="https://www.ncbi.nlm.nih.gov/clinvar/RCV003152766.1/" data-mce-href="https://www.ncbi.nlm.nih.gov/clinvar/RCV003152766.1/">RCV003152766.1	href="https://www.ncbi.nlm.nih.gov/clinvar/RCV003152766.1/" data-mce-href="https://www.ncbi.nlm.nih.gov/clinvar/RCV003152766.1/">RCV003152766.1	NA	NA	href="https://www.ncbi.nlm.nih.gov/clinvar/RCV002287504.2/" data-mce-href="https://www.ncbi.nlm.nih.gov/clinvar/RCV002287504.2/">RCV002287504.2	NA

Patient 1 exhibited skewed X-chromosome inactivation (15:85; Diagnostic Genomics, PathWest, Western Australia). In Individual 1 and 2, X-inactivation was random and 98% skewed, respectively [22], both accompanied by severe clinical presentations. These findings suggest an X-linked dominant mode of inheritance, in which a single mutated *MED12* allele is sufficient to cause disease. Because Patient 1’s presentation mirrors that of the six previously reported cases, further investigation into the underlying disease mechanism is warranted.

### Patient-derived iPSCs with expression of healthy or variant MED12

We derived iPSCs from patient peripheral blood mononuclear cells (PBMCs) to investigate mechanisms of disease and characterise altered biological pathways associated with the *MED12* VUS. Two clonal iPSC lines were selected for further study based on quality assessment. Targeted amplicon sequencing (> 40,000 reads/cell line) confirmed the heterozygous *MED12* c.3412 C > T; p.Arg1138Trp variant (Fig. 1C, D). Because *MED12* resides on the X chromosome, we examined allelic expression using targeted amplicon sequencing of cDNA (> 20,000 reads per line) derived from whole-cell RNA. The two clones showed differential X-inactivation, expressing either WT *MED12* transcript or the *MED12* c.3412 C > T; p. Arg1138Trp transcript (Fig. 1C, D). Hereafter, these cell lines are termed MED12_WT and MED12_VUS, respectively. We assessed nuclear localisation of MED12 by immunofluorescence. In both MED12_WT and MED12_VUS iPSCs, the MED12 protein localised to the nucleus (Fig. 1E).

### Directed differentiation of patient iPSCs to neural progenitor cells

**Fig. 2:**
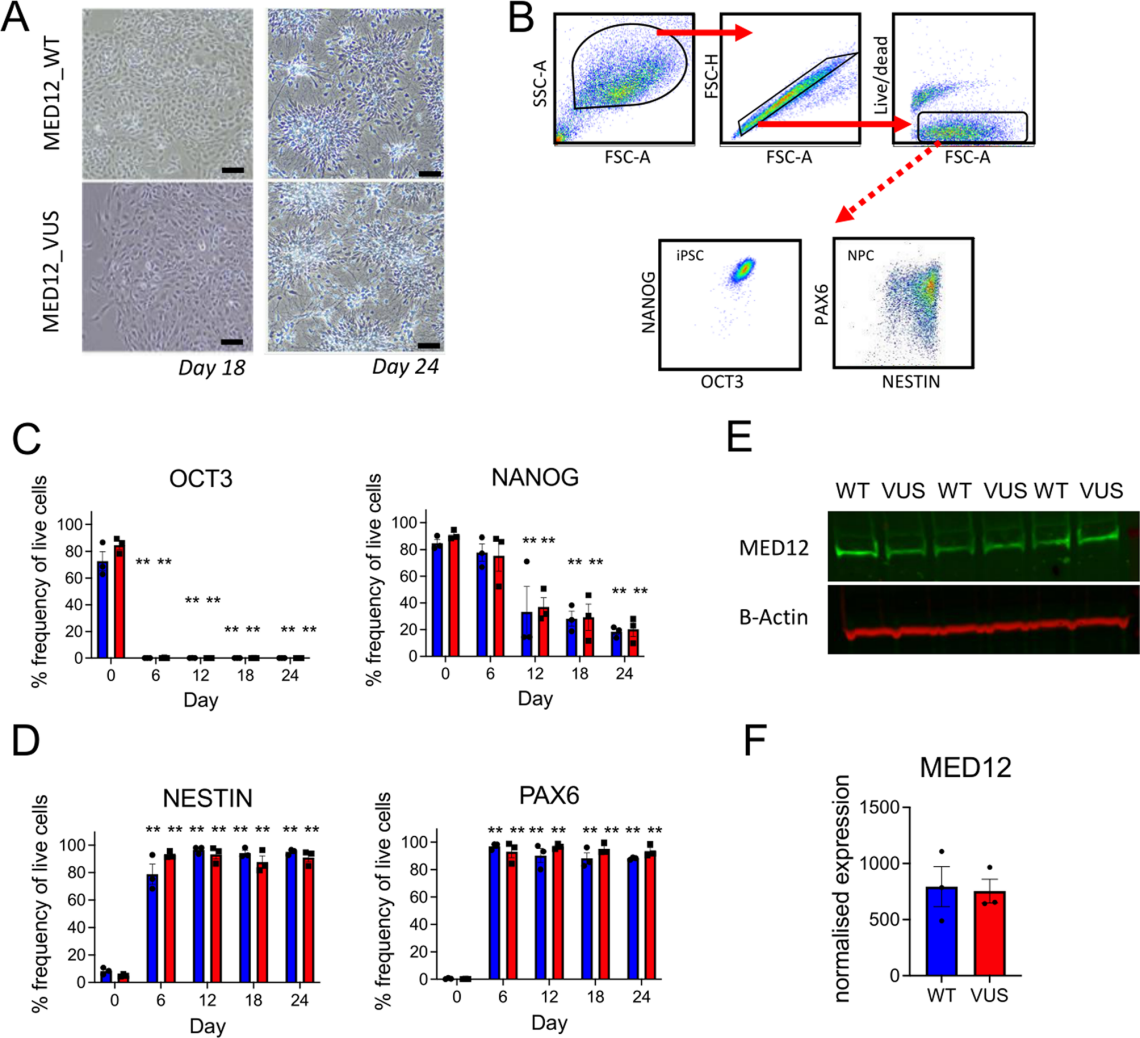
Neural disease modelling for MED12_WT and MED12_VUS. iPSCs were induced to differentiate into neural progenitor cells and examined for changes in morphology, marker expression, and MED12 protein levels at indicated timepoints. **A** Light-microscopy images of NPCs at days 18 and day 24 showing bipolar cells with long dendrites. **B** Flow cytometry gating strategy for iPSCs and NPCs during neural cell differentiation. **C and D** Bar graphs show the percentage of live cells expressing stem or neural markers, respectively. Timepoints as indicated. MED12_WT (blue), and MED12_VUS, (red). Mixed-model two-way ANOVA with Bonferroni’s multiple comparison test. (*n* = 3 group). *p < 0.05; **p < 0.01. **E** MED12 western blot in NPCs at day 24 of neural cell differentiation, and **F** Bar graph indicates MED12 protein expression, normalized to β- Actin expression, in MED12_WT and MED12_VUS NPCs

As the patient presented with a neural-related disease phenotype, we differentiated the iPSCs to neural progenitor cells and assessed cell morphology, and expression of stem and neural markers. In MED12_WT and MED12_VUS we observed normal cell morphology at day 18 and 24, with formation of elongated bi-polar cells with long dendrites by day 24 (Fig. 2A). Stem cell markers decreased in both lines, whereas the neural markers NESTIN and PAX6 were upregulated during differentiation, as expected (Fig. 2C, D; Suppl. Figure 2 A). MED12 protein levels were similar in both cell lines after differentiation (Fig. 2E, F; Suppl. Figure 2 C). Taken together, these findings confirm normal differentiation of MED12_WT and MED12_VUS iPSCs into NPCs. Additionally, amplicon sequencing across the variant region in NPC cDNA confirmed that the pattern of X-inactivation was maintained for both cell lines after differentiation (Suppl. Figure 2B).

### Transcriptomics analysis of MED12_WT and MED12_VUS neural differentiation

**Fig. 3:**
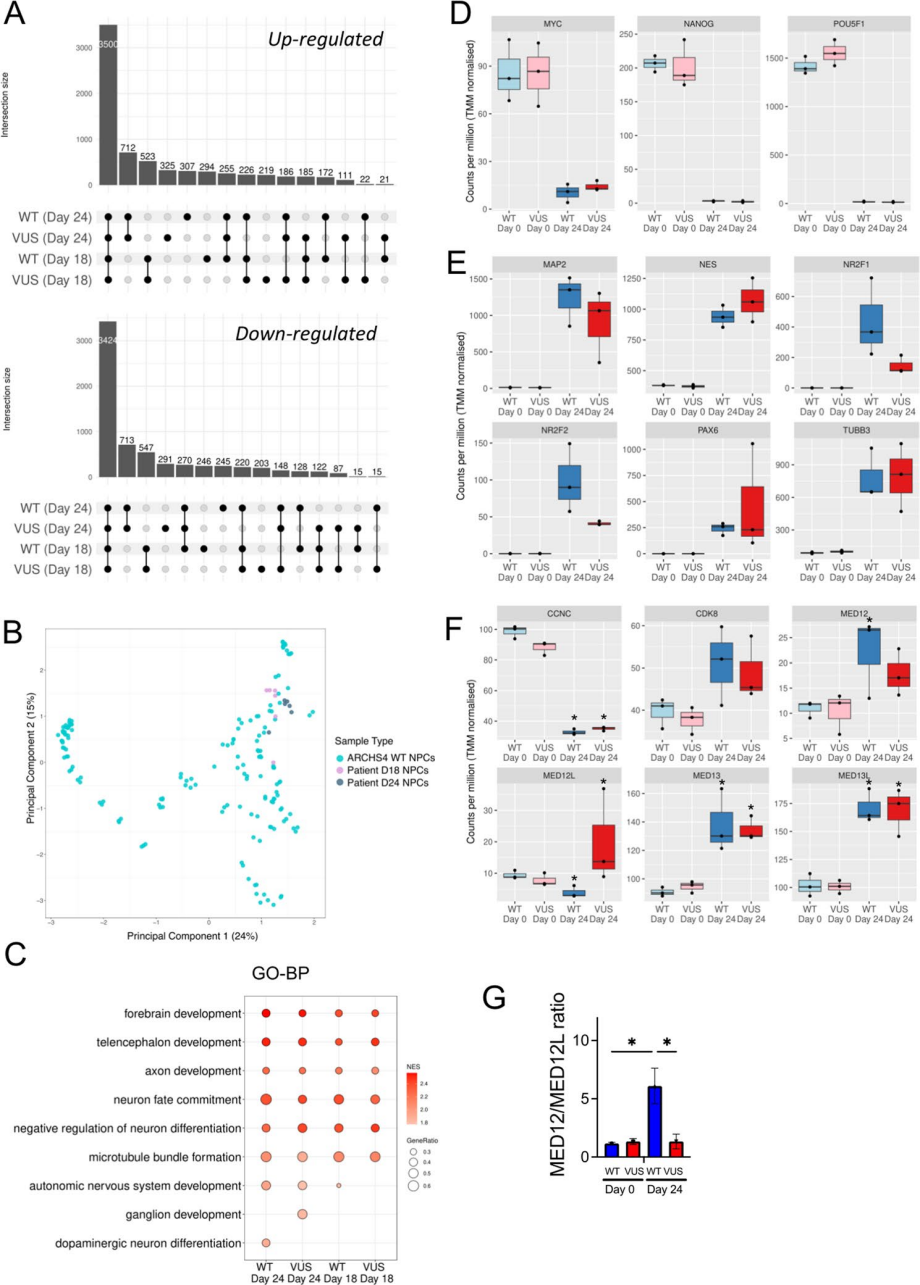
Changes in MED12 and components of the MKM during neural cell differentiation. MED12_WT and MED12_VUS iPSCs were stimulated for neural cell differentiation for transcriptomics analysis. **A** Upset plots showing DEGs common to WT and VUS differentiation at days 18 and 24. **B** Comparison of NPCs to public NPCs sourced from the ARCHS4 dataset. **C** GSEA GO-BP geneset enrichment indicates upregulation of neural pathways at day 18 and day 24 in NPCs compared to respective iPSCs. **D and E** Box plots indicate down-regulation of stem cell markers at and up-regulation of neural cell markers at day 24. **F** Box plots indicate changes in transcript expression for components of the MKM. **G** Bar graph shows the change in the MED12/MED12L ratio during differentiation. Boxplots adjusted p-value < 0.05; Bar graph, One tailed, unpaired t-test, *p* < 0.05

We compared NPCs to iPSCs for MED12_WT and MED12_VUS neural cell differentiation to identify differentially expressed genes (Fig. 3A; Suppl. Table 3) and contrasted patient-derived cells at days 0, 18, and 24 of neural cell differentiation. To assess whether our NPCs show global differences in gene expression compared to normal NPCs, we compared their transcriptome to reference transcriptomes deposited on the ARCHS4 database using Principal Component Analysis. All NPCs clustered tightly together, and we conclude that there are no major global changes (Fig. 3B). To further investigate whether normal differentiation took place, we performed pre-ranked Gene Set Enrichment Analysis (GSEA) comparing NPCs to their respective iPSCs and confirmed upregulation of classical neuronal developmental pathways (Fig. 3C; Suppl. Table 4). As expected, and in alignment with our observations at the protein level, standard differential gene expression analysis showed a consistent down regulation of stem and upregulation of neuronal markers (Fig. 3D, E). These results provide additional evidence for the differentiation of our iPSC lines into neural progenitor cells.

### Changes in the mediator kinase module

Gene expression of the Mediator Kinase Module (MKM) components (*MED13*, *MED13L*, *CDK8*, *CCNC*, *MED12* and *MED12L*) during neural differentiation was investigated using the RNA sequencing data. In NPCs compared with iPSCs, *MED13* and *MED13L* (the paralog of *MED13*) were expressed at significantly higher levels, whereas *CCNC* expression was significantly reduced and *CDK8* levels were not significantly different (Fig. 3F; Suppl. Table 3). In MED12_WT NPCs, *MED12* expression increased significantly while *MED12L* decreased (Fig. 3F), resulting in higher *MED12*/*MED12L* ratio (Fig. 3G). Conversely, in MED12_VUS NPCs, *MED12* was not upregulated and *MED12L* expression was significantly increased. The results indicate significant different in the expression of *MED12* and *MED12L* components of the MKM in MED12_WT and MED12_VUS NPCs.

### Changes in neural gene expression in comparison of MED12 p.Arg1138Trp to WT NPCs

Comparing MED12_VUS with MED12_WT during neural differentiation, transcriptomic analysis identified 25 differentially expressed genes (DEGs) at day 18 and 51 DEGs at day 24, with 19 DEGs in common (Suppl. Table 5). At day 24, the top five significantly upregulated genes in MED12_VUS NPCs were *F8A2*, *F8A3*, *PCDHA6*, *RGPD1*,and *ADPRHL1*. *F8A2* and *F8A3* are involved in endosome motility, while *PCDHA6* is associated with the establishment and maintenance of neuronal connections. *RGPD1* is involved in import of nuclear localisation signal bearing proteins into the nucleus and linked to cerebral folate deficiency [25], which may be relevant to the patient phenotype of athetoid cerebral palsy.

The top five significantly downregulated genes at day 24 were *PEG3*, *MIMT1*, *ZIM2*, *PCDHGA3*, and *EOMES*. *PEG3* and *ZIM2* are associated with non-syndromic X-linked intellectual disability 2, RNA polymerase II-specific DNA binding and neuronal cell death, whereas *PCDHGA3* and *EOMES* are implicated in brain development. Notably, seven of the 51 DEGs at day 24 are directly linked to *POLR2A*. Specifically, *ZIM2*, *ZFP3*, *TBR1*, *PEG3*, *EMX1*, and *DMRTA2* were downregulated, while the *POLR2A* repressor *ZNF558* was upregulated. Furthermore, several downregulated genes relate to brain development and intellectual disability, including *BMP3*, *EBF2*, *EMX1*, *EOMES*, *LXH5*, *NHLH1*, and *UNCX*. In addition, neural-related genes that were significantly upregulated include *CNP*, which is involved in neurogenesis; *TCF7L2*, which plays a role in WNT signalling during neural cell differentiation; and *ZDBF2*, which is regulated during the development of the hypothalamic-pituitary-adrenal axis. Overall, the data indicate that the MED12 p.Arg1138Trp mutation in NPCs alters gene expression in a manner that may contribute to dysfunction of *POLR2A* transcription, neural development, and neural cell connections.

### Changes in SHH and WNT pathways relevant to MED12 protein domains

We next investigated cellular pathways associated with MED12’s two characterised functional domains (Fig. 1A): the MED12 domain and the Med12-PQL domain. The MED12 domain is essential to the MKM and interacts with Gli3 in RNA Polymerase II to regulate the Gli3-dependent Sonic Hedgehog (SHH) signalling pathway [26], whereas the Med12-PQL domain binds β-catenin to activate transcription of Wnt-responsive genes [27]. GSEA analysis contrasting our differentiated cell lines revealed significant changes in Gli3 (SHH)- and WNT-related gene sets as well as enrichments for genes involved in axon development, forebrain development, pattern specification process, regionalization, neural precursor cell proliferation, and telencephalon development (Suppl. Figure 4 A, B; Suppl. Table 6). These data indicate persistent downregulation of SHH-related neural pathways in MED12___VUS compared to MED12_WT NPCs, likely reflecting dysregulation in neural cell development and specification.

### Differences in neural development in MED12 variant NPCs

**Fig. 4:**
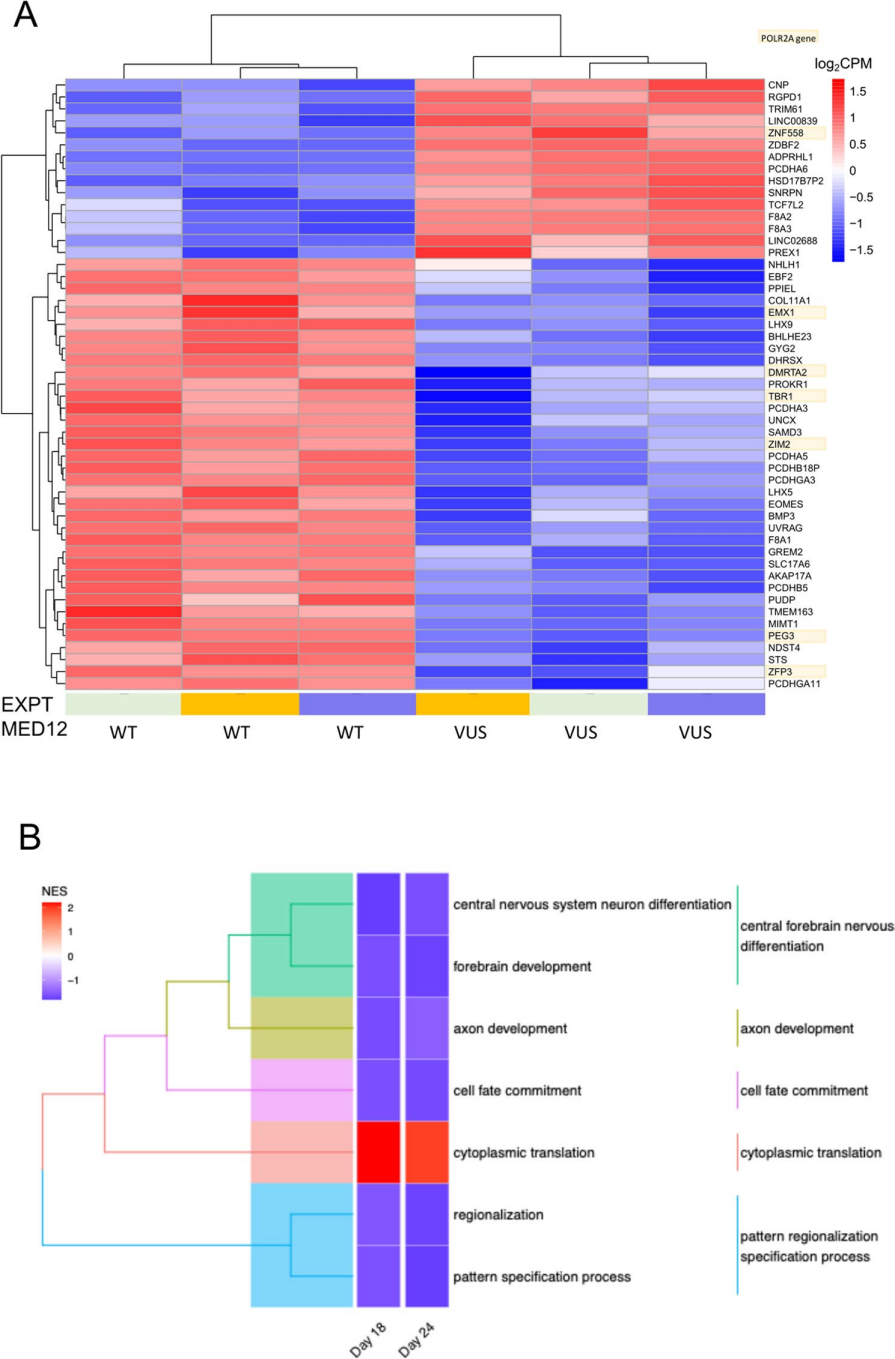
MED12 variant alters gene expression and neural development. MED12_WT and MED12_VUS iPSCs were differentiated into NPCs and transcriptomic profiles of MED12_VUS and MED12_WT NPCs were compared. **A** Heatmap of top differentially expressed genes. **B** Treeplot of GO-BP terms enriched when comparing NPCs. Colour indicates normalised enrichment score, representing overall direction of enrichment. Red (positive) represents overall up-regulation, blue (negative) represents overall down-regulation

**Fig. 5:**
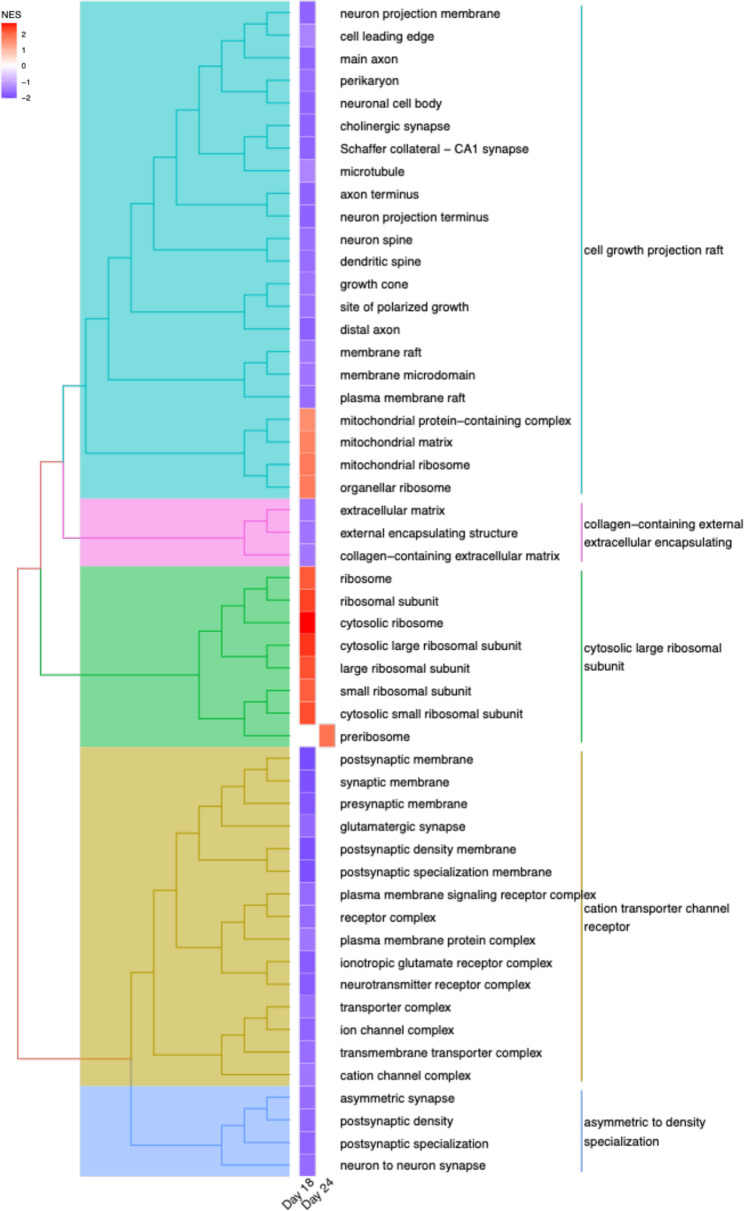
Neural cells carrying the MED12 variant show altered cell growth, specification, and ribosomal complex formation. The MED12_WT and MED12_VUS iPSCs were stimulated for neural cell differentiation, and transcriptomics performed using GSEA and the GO-CC data set. Treeplot demonstrates enriched GO-CC terms when comparing MED12_VUS NPCs to MED12_WT NPCs at days 18 and 24. Terms are clustered based on similarity in gene set. Colour indicates direction of enrichment, with red representing overall upregulation of gene set, blue representing overall downregulation

To further investigate changes in biological processes and cellular components, we visualised significant terms in tree plots of GSEA results for GO Biological Process (GO-BP) and GO Cellular Component (GO‐CC) gene sets. In the GO‐BP analysis, gene sets significantly up-regulated in MED12_VUS relative to MED12_WT included cytoplasmic translation (Fig. 4B; Suppl. Table 6). Conversely, down-regulated gene sets were associated with neural development, including central forebrain nervous system differentiation, axon development and cell specification. In the GO‐CC analysis, temporal changes in neural cell development were observed between wildtype and VUS NPCs (Fig. 5; Suppl. Table 7). At day 18, gene sets linked to structural components required for axonal growth and neurotransmission were significantly altered. In addition, gene sets related to cation transporter channels and the specialisation of asymmetric neuron synapses were downregulated. Ribosomal‐related gene sets were significantly upregulated at day 18, consolidating to a single pre‐ribosome gene set by day 24. Overall, these findings indicate temporal changes in neural cell development in MED12_VUS cells, with a notable increase in cytoplasmic translation and ribosome function.

## Discussion

The mediator complex is involved in the interaction between transcription factors and RNA polymerase II to modulate transcription initiation. MED12, a subunit of this complex, regulates this process as part of the MKM. This study presents the first functional analysis of the MED12 p.Arg1138Trp variant, previously reported in six other female individuals with variable clinical presentations. We modelled the disease by differentiating patient-derived iPSCs into neural cells. Expression of MED12 p.Arg1138Trp in iPSC-derived NPCs altered development and function, inducing temporal changes in neural specification, dysregulation of the MKM components, and increased ribosomal activity.

Phenotypic comparison of seven females harbouring the MED12 p.Arg1138Trp variant revealed diverse clinical presentations. All individuals presented with severe intellectual disability, behavioural abnormalities (autism, social, attention span, global developmental delay), ophthalmologic anomalies, skull anomalies, gut anomalies (constipation, dysmotility, vomiting), and delayed or absent speech. Such variability likely stems from MED12’s central cellular role, and the Mediator complex’s interaction with more than 3,000 transcription factors [2, 22–24]. Accordingly, MED12 variants may underlie numerous syndromes, and the current catalogue of MED12-related disorders is likely incomplete [28, 29].

Analysis of MED12_WT and MED12_VUS neural differentiation revealed significant pathway changes in forebrain development, axonogenesis, cell specification, and synapse formation. Notably, MED12_VUS cells exhibited delayed neural maturation and specification. Previous work shows that the Mediator complex stabilises enhancer-promoter loops and modulates transcription factor activity [1, 30]. MED12 also clusters with active histone marks (H3K27ac, H3K4me3) and pluripotency factors (Oct4, Sox2, Nanog) to regulate gene expression in mouse ESCs [31]. The *MED12* variant in patient-derived cells may lead to dysregulation of the euchromatin state and disrupt maintenance of pluripotency factors, ultimately delaying neural cell maturation.

The MKM regulates the Mediator complex. Strikingly, cells carrying the *MED12* variant displayed altered expression of both *MED12* and its paralogue *MED12L*, which are mutually exclusive within the MKM. In variant cells, *MED12L* is up-regulated during neural differentiation, whereas WT cells up-regulate *MED12* and down-regulate *MED12L*. This suggests that *MED12L* may compensate for reduced *MED12* function. Other studies indicate that MED12L and MED12 are mutually exclusive in forming the mediator kinase module [1]. Our finding is consistent with Med12l upregulation observed in *Med12* mutant mouse ESCs [24]. Consistently, *Med12* mutant mouse ESCs show *Med12*l up-regulation accompanied by impaired maturation and development [32]. Preferential MED12L incorporation into the MKM may therefore perturb Mediator complex, RNA polymerase II activity, and neural development.

MED12 interacts with Gli3 via RNA polymerase II to regulate the Sonic Hedgehog (SHH) pathway [26]. Studies indicate that dysregulation of the SHH signalling pathway and downregulation of GLI3 pathways result in impaired brain development [33, 34]. Here, we identified SHH-related alterations in neural development that align with the Patient 1 phenotype of hydrocephaly, epilepsy and cerebral palsy. The MED12_VUS NPCs exhibit alterations in telencephalon development pathways. Interestingly, suppression of telencephalon development is closely linked to hydrocephaly [35]. Other cellular pathway changes in MED12_VUS were in forebrain development, where changes in development are a known cause of epilepsy and cerebral palsy [36].

Finally, the nucleolus is the site of rRNA gene transcription by RNA polymerase I, ribosome biogenesis, and export of ribosomes to the cytoplasm to support protein synthesis [37]. In genetic variant cells, we observed up-regulation of pre-ribosome pathways and increased cytoplasmic translation. Because nuclear sequestration of proteins is involved in chromatin regulation and gene expression [2, 32, 37], the *MED12* variant protein may disrupt this process. Indeed, others report that dysregulation of *MED12L* is linked to intellectual disability in Nizor-Isidor Syndrome (OMIM 618872) and associated with transcriptional defects [38]. Furthermore, recent MED12 knockdown experiments in *Drosophila* indicate a role for *MED12* in regulation of ribosome pathways [39].

## Conclusions

This study offers the first functional evidence for MED12 p.Arg1138Trp using iPSCs directed towards neural differentiation. Expression of MED12 p.Arg1138Trp caused temporal changes in neural development, and increased ribosome biogenesis, implicating MKM and Mediator complex components in disease aetiology. A key limitation of the current study is the use of an immature NPC model to recapitulate the disease phenotype. Additional mechanistic insights might be gained using mature neuron or cerebral organoid models. Nonetheless, the functional differences observed in NPCs expressing MED12 p.Arg1138Trp suggest that the associated clinical phenotype arises early in neural development.

## Supplementary Information

Supplementary Material 1. Supplementary fig. 1. Assessment of patient-derived iPSCs Patient iPSC clones WT and VUS were assessed for clearance of the reprogramming vectors, cell morphology, karyotype, and trilineage capacity. **(A)** Bar graph shows clearance of the SeV, KOS, KLF4, and c-Myc reprogramming vectors as determined by qPCR. **(B)** Light microscopy images show typical stem cell morphology scale bar, 100 μm. **(C)** Karyotype integrity was confirmed by qPCR for common amplifications and deletions in iPSCs. **(D)** MED12_WT and MED12_VUS iPSCs were used in a trilineage assay and assessed for expression of the stem, endoderm, mesoderm and ectoderm markers by flow cytometry. Gating strategy as per Fig. 2B. Histogram plot, left, indicates expression of the stem cell marker OCT3. Dot plots indicate the expression of endoderm cell markers (blue dots; SOX17, CXCR4), mesoderm (red dots; Brachyury, CXCR4) and ectoderm (purple dots; PAX6, Nestin) as determined following trilineage directed differentiation. Supplementary fig. 2. Characterization of MED12_WT and MED12_VUS cells during neural progenitor cell differentiation iPSCs were stimulated for neural progenitor cell differentiation and examined for changes in cell morphology, stem and neural marker expression, and MED12 protein expression at indicated timepoints. **(A)** Flow cytometry analysis of live cells gated according to Fig. 2B. Bar graphs show the mean fluorescence intensity (MFI) of stem cell markers (OCT3, NANOG) and neural markers (PAX6, Nestin) in live cells. WT (blue) and MED12 variant (red). Mixed-model two-way ANOVA with Bonferroni’s multiple comparison test. (*n* = 3 group). *p < 0.05; **p < 0.01. **(B)** MED12 targeted amplicon sequencing on iPSC-derived NPCs indicating maintenance of X-chromosome inactivation (> 1300 reads/sample). **(C)** Full western blot image for MED12 expression in NPCs at day 24. Supplementary fig. 3 Principal component analysis (PCA) of iPSCs and NPCs. Plots demonstrate separation of iPSCs from NPCs at day 18 and day 24, as indicated. Supplementary fig. 4. Changes in Gli and WNT related pathway gene-sets GSEA GO-BP significant gene-sets in the comparison of MED12_VUS to MED12_WT NPCs at day 18 and day 24 of neural cell differentiation. **(A)** Gli related pathways, and **(B)** WNT related pathways.

Supplementary Material 2. Supplementary table 1 Geneticist clinician reported HPO terms for Patient 1. Supplementary table 2 MED12 Patient 1 HPO terms and MED12 related disorders. Supplementary table 3 Differentially expressed genes NPCs compared to iPSCs. Supplementary table 4 GSEA GO-Biological Processes, Neural differentiation of patient-derived iPSCs. Supplementary table 5 Differentially expressed genes in comparison of variant to WT NPCs. Supplementary Table 6. GSEA GO_BP in difference of NPCs. Supplementary Table 7. GSEA GO_CC in difference of NPCs.

## Data Availability

Datasets are grouped under the GSE288407 SuperSeries as two different GEO datasets: GSE288302 for RNASeq, and GSE288303 for AmpSeq.

## Abbreviations

DEG: Differentially expressed genes
GO: Gene Ontology
GO-CC: GO-Cellular component
GO-BP: GO-Biological Processes
GSEA: Gene Set Enrichment Analysis
iPSCs: Induced pluripotent stem cells
HS: Hardikar syndrome
FGS: FG syndrome
LS: Lujan syndrome
MKM: Mediator Kinase Module
NPCs: Neural progenitor cells
PCA: Principal Component Analysis
VUS: Variant of uncertain significance
XLOS: X-linked Ohdo syndrome
