# Supplementary figures and images for: Functional characterization of the MED12 p.Arg1138Trp variant in females: implications for neural development and disease mechanism
Source: Mol Med. 2025 Sep 29;31:300. doi: 10.1186/s10020-025-01365-5 (PMC12482371; doi:10.1186/s10020-025-01365-5)

Supp Fig 1.

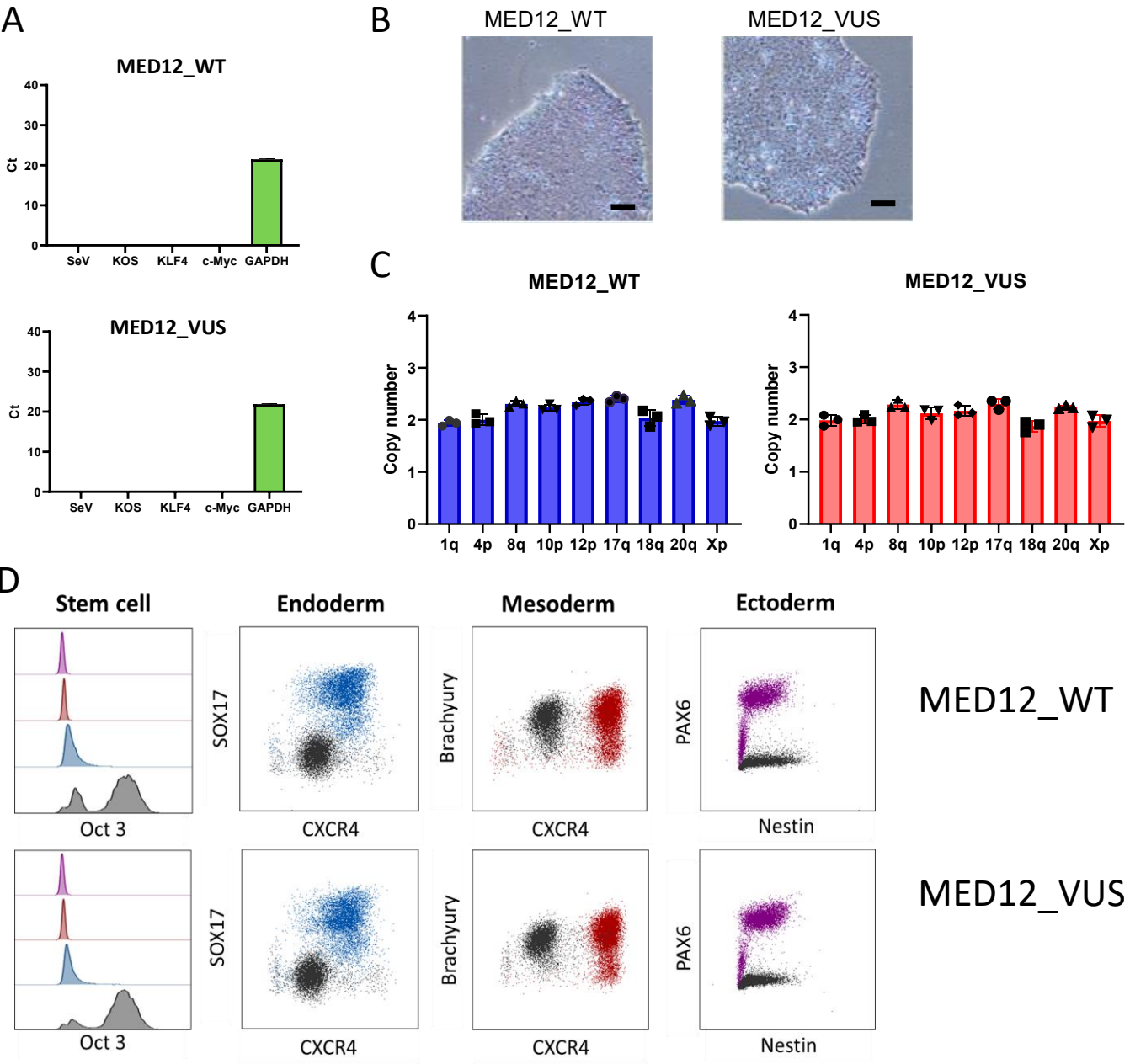

Supp Fig 2.

A

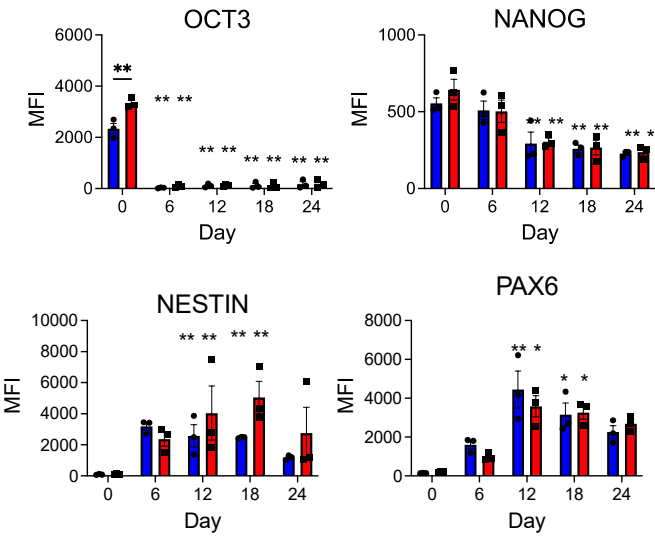

B

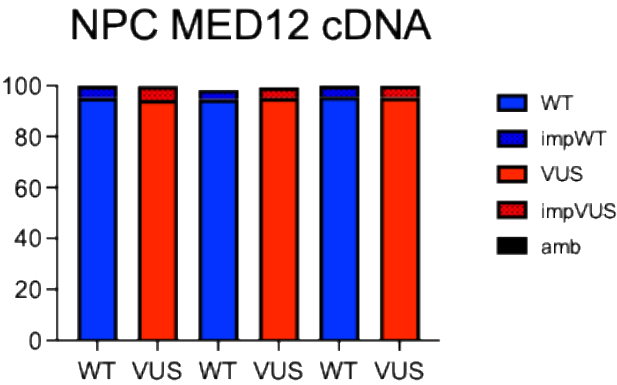

C

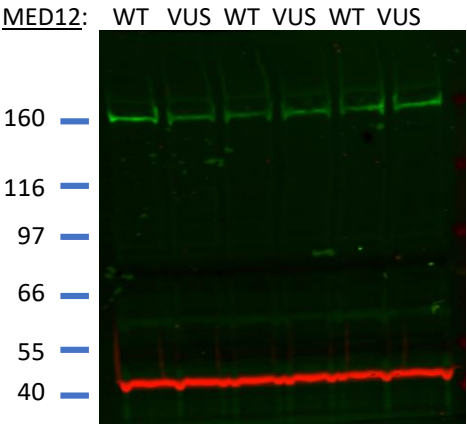

Supp Fig 3.

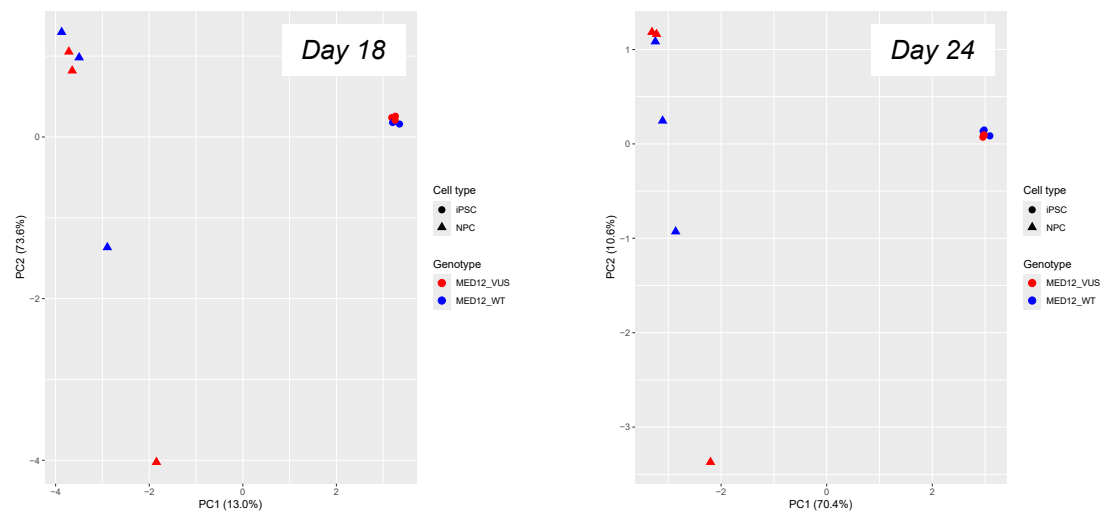

Supp Fig 4.

A

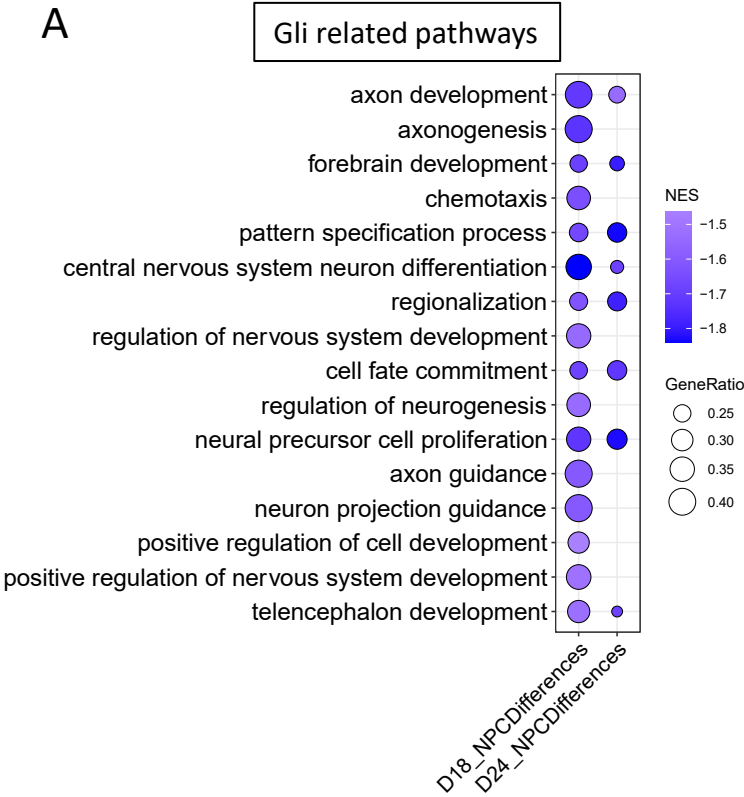

B

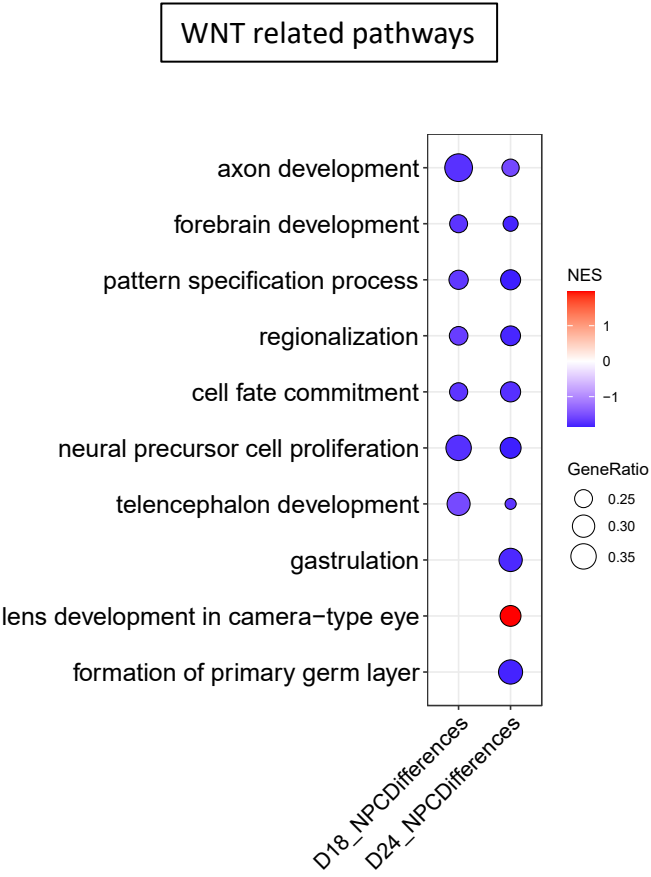

Supplement: Supplementary file 1 — Supplementary Material 1. Supplementary fig. 1. Assessment of patient-derived iPSCs Patient iPSC clones WT and VUS were assessed for clearance of the reprogramming vectors, cell morphology, karyotype, and trilineage capacity. (A) Bar graph shows clearance of the SeV, KOS, KLF4, and c-Myc reprogramming vectors as determined by qPCR. (B) Light microscopy images show typical stem cell morphology scale bar, 100 μm. (C) Karyotype integrity was confirmed by qPCR for common amplifications and deletions in iPSCs. (D) MED12_WT and MED12_VUS iPSCs were used in a trilineage assay and assessed for expression of the stem, endoderm, mesoderm and ectoderm markers by flow cytometry. Gating strategy as per Fig. 2B. Histogram plot, left, indicates expression of the stem cell marker OCT3. Dot plots indicate the expression of endoderm cell markers (blue dots; SOX17, CXCR4), mesoderm (red dots; Brachyury, CXCR4) and ectoderm (purple dots; PAX6, Nestin) as determined following trilineage directed differentiation. Supplementary fig. 2. Characterization of MED12_WT and MED12_VUS cells during neural progenitor cell differentiation iPSCs were stimulated for neural progenitor cell differentiation and examined for changes in cell morphology, stem and neural marker expression, and MED12 protein expression at indicated timepoints. (A) Flow cytometry analysis of live cells gated according to Fig. 2B. Bar graphs show the mean fluorescence intensity (MFI) of stem cell markers (OCT3, NANOG) and neural markers (PAX6, Nestin) in live cells. WT (blue) and MED12 variant (red). Mixed-model two-way ANOVA with Bonferroni’s multiple comparison test. (n = 3 group). *p < 0.05; **p < 0.01. (B) MED12 targeted amplicon sequencing on iPSC-derived NPCs indicating maintenance of X-chromosome inactivation (> 1300 reads/sample). (C) Full western blot image for MED12 expression in NPCs at day 24. Supplementary fig. 3 Principal component analysis (PCA) of iPSCs and NPCs. Plots demonstrate separation of iPSCs from NPCs [file 10020_2025_1365_MOESM1_ESM.pdf]
